# Intimal thickening and disruption of the media occur in the arterial walls of coronary arteries not associated with coronary arterial aneurysms in patients with Kawasaki disease

**DOI:** 10.1186/s12872-021-02090-7

**Published:** 2021-06-05

**Authors:** Tomoya Tsuchihashi, Nobuyuki Kakimoto, Takashi Takeuchi, Tomohiro Suenaga, Takayuki Suzuki, Shoichi Shibuta, Yasushi Ino, Takashi Kubo, Takashi Akasaka, Hiroyuki Suzuki

**Affiliations:** 1 Department of Pediatrics, Wakayama Medical University, 811-1 Kimiidera, Wakayama, 641-0012 Japan; 2 Department of Pediatrics, Kinan Hospital, 46-70 Shinjo-cho, Tanabe, Wakayama, 646-8588 Japan; 3 Department of Cardiovascular Medicine, Wakayama Medical University, 811-1 Kimiidera, Wakayama, 641-0012 Japan

## Abstract

**Background:**

Coronary artery aneurysm (CAA) is an important complication of Kawasaki disease (KD) that is associated with arterial structure damage. However, few studies have examined structural changes in coronary arteries that are not associated with CAA.

**Methods:**

We examined coronary arteries in KD patients with CAAs who underwent follow-up coronary angiography (CAG) and optical coherence tomography (OCT). Coronary arterial branches with no abnormal findings during the most recent CAG were classified into two groups. Arteries with an acute-phase CAA that later regressed were classified as group R; arteries with no abnormal findings on either acute or convalescent phase CAG were classified as group N. Coronary arterial wall structural changes were compared between groups using OCT.

**Results:**

Fifty-seven coronary arterial branches in 23 patients were evaluated by OCT. Thirty-six branches showed no abnormality during the most recent CAG. Both groups R and N comprised 18 branches. Maximum intimal thicknesses in groups R and N were 475 and 355 µm, respectively (p = 0.007). The incidences of media disruption were 100% and 67%, respectively (p = 0.02). Calcification, macrophage accumulation, and thrombus were not found in either group.

**Conclusions:**

Intimal thickening and disruption of the media occur in coronary arteries with acute phase CAAs that later regress in the convalescent phase, as well as in arteries with normal CAG findings in the acute and convalescent phases.

## Background

Kawasaki disease (KD) is an acute systemic vasculitis that appears most frequently in infants and children. Because treatment has improved with intravenous immunoglobulin (IVIG) and other therapies (e.g., steroids [1], infliximab [2], and cyclosporine [3]), the incidence of coronary artery aneurysm (CAA) has decreased to 2.6% according to a 2017–2018 nationwide survey in Japan [4]. However, the absolute number of patients who develop CAA in Japan has not substantially changed because the incidence of KD has been increasing. Therefore, preventing CAA development remains a major clinical concern.

Most small CAAs (i.e., smaller than medium size) in the acute phase of KD undergo regression in the convalescent phase. On ultrasound cardiography (UCG) and selective coronary angiography (CAG), these arteries revert to a normal appearance. However, the abnormal vascular structure is present at the previous site of an acute CAA because of healing-related intimal thickening [5]. Although many autopsy reports have described structural changes within a dilated coronary artery [5, 6], only a few reports have described in vivo coronary arterial wall changes in CAAs using intravascular ultrasound (IVUS) and optical coherence tomography (OCT) [7–9]. In addition, few reports have evaluated normal coronary arterial branches without CAAs following the acute phase in KD patients evaluated by OCT [9].

Although IVUS has been used to evaluate coronary arterial walls, detailed evaluation is difficult because of its poor resolution (100–200 µm). OCT is a newer intravascular imaging modality that can be used as an alternative to IVUS [10–12]. OCT has a higher resolution (10–20 µm) and can clearly discriminate the three laminar structures (intima, media, and adventitia) of vessel walls. Furthermore, it can clearly differentiate tissue properties (e.g., calcification, adipose tissue, and macrophage colonization) within vessel walls.

This study used OCT to investigate arterial wall structures in CAA-containing branches in the acute phase, which subsequently regressed in the convalescent phase, in KD patients with CAAs. It also investigated arterial wall structures in non-CAA-containing coronary branches after the acute phase in KD patients with CAAs, then compared arterial wall structures between the two types of branches.

## Patients and methods

### Patients

This retrospective study enrolled KD patients who developed a CAA (inner coronary artery diameter ≥ 4 mm) on UCG at 1 month after disease onset. CAAs were defined in accordance with the 1983 Japanese Ministry of Welfare criteria [13] and confirmed by CAG, which was performed repeatedly in accordance with KD guidelines [14]. All patients regularly underwent UCG at our hospital; Z-scores of coronary artery diameters were retrospectively calculated using the Z-score calculator (Version 4.0 Full, LMS_Z_Score). In follow-up CAG performed between January 2012 and March 2018*,* OCT was performed concurrently in patients with bodyweight ≥ 30 kg. The study was approved by the Wakayama Medical University Ethical Review Board (No. 2916) and adhered to the ethical guidelines of the 1975 Declaration of Helsinki. Written informed consent was obtained from all participants.

### CAG

Bilateral selective CAG was performed. After a 6-Fr sheath had been placed in the right radial artery, 5-Fr catheters were advanced to the coronary ostia bilaterally for contrast injection.

### OCT procedure

For OCT, a 6-Fr guiding coronary artery catheter was placed at the origin of each coronary artery to advance a guidewire. A C7 Dragonfly Intravascular Imaging Catheter (0.036-in. outer diameter; St. Jude Medical, Inc., St. Paul, MN, USA) was inserted into the distal coronary artery. The guiding catheter was continuously flushed with contrast medium to remove blood cells from the observation region during OCT scanning. OCT images were collected using an automatic pullback device (20 mm/s) at a frame rate of 100 frames/s and recorded on a C7-XR OCT Intravascular Imaging System (St. Jude Medical, Inc.).

### Evaluation of coronary arterial wall structure changes

In coronary arteries in which OCT could be applied, we focused on branches without dilatation or stenosis on the most recent CAG. The branches were classified into two groups based on CAG findings. Branches that developed CAA in the acute phase and later regressed in the convalescent phase (“normalization”) were classified as group R. Branches with no abnormal findings in either the acute or convalescent phases were classified as group N. We excluded branches with residual CAA on the most recent CAG from this study, because it is difficult to accurately evaluate the entire circumference of the CAA in these branches on OCT because of insufficient blood cell washout with contrast medium and/or inadequate observational range.

In group R, OCT images of a 10-mm segment of the region that included the acute-phase CAA were extracted at 1-mm intervals. In group N, images were extracted from a 10-mm segment of the origin of each branch, where CAA had never developed. We excluded branches that had imaging artifacts or included a side branch that comprised > 25% of the observed region.

### Analysis of OCT images

**Fig. 1:**
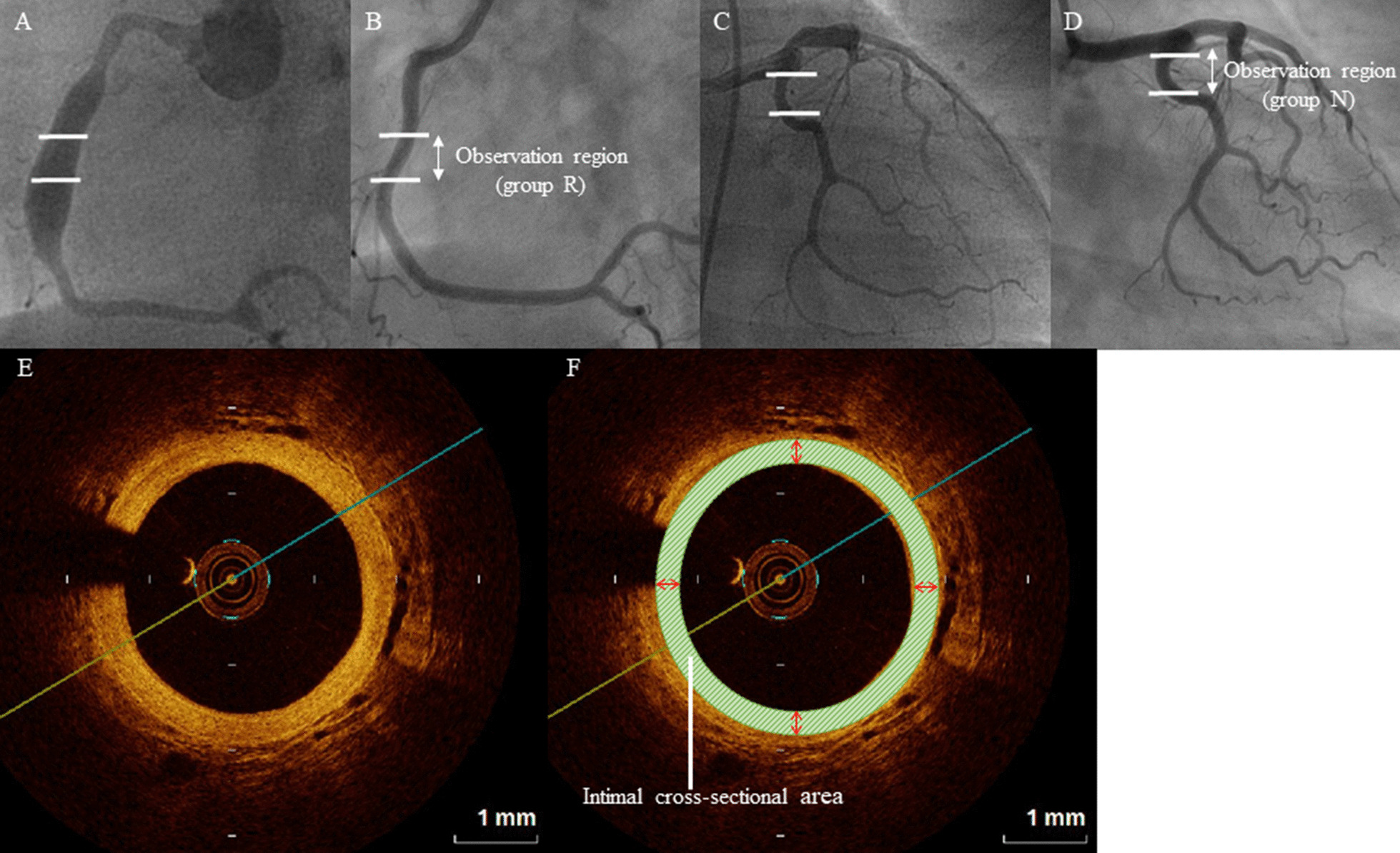
**A**, **B** Right coronary angiography (CAG). **C**, **D** Left CAG. **E**, **F** Optical coherence tomography (OCT) coronary artery images. (**A**, **B**) In group R, we evaluated a 10-mm coronary arterial segment that developed CAA in the acute phase (**A**) and regressed in the convalescent phase (**B**). (**C**, **D**) In group N, we evaluated a 10-mm segment of the origin of each branch, where there were no abnormal changes in the acute (**C**) or convalescent (**D**) phases. **E** OCT image showing disruption of the media and abnormal intimal thickening. **F** We calculated the intimal cross-sectional area by subtracting the medial area (i.e., vascular lumen cross-sectional area) from the circumferential medial cross-sectional area; the mean area of the target cross sections was determined

All OCT imaging data were digitized, transferred to ImageJ (US National Institutes of Health, Bethesda, MD, USA), and analyzed in two-dimensions by two observers who were blinded to CAG findings. OCT imaging of the three laminar areas was acquired in a concentric pattern from the vascular lumen to the outside, thus showing the intima, media, and adventitia. Maximum intimal diameter was measured. Intimal cross-sectional area was calculated by subtracting the medial area (i.e., vascular lumen cross-sectional area) from the circumferential medial cross-sectional area; the mean area of the target cross sections was determined (Fig. 1).

In previous studies, coronary artery intimal thickness < 300 µm was considered normal [7, 16–18]; therefore, we defined thickness > 400 µm as abnormal. In addition to intimal thickness and cross-sectional area, the following parameters were evaluated and compared between groups R and N: disruption of the media, calcification (sharply delineated borders with heterogeneous poor signal composition), macrophage colonization (signal-rich, distinct, and greatly attenuated OCT light), and thrombus (mass attached to the luminal surface or floating within the lumen). All evaluations were made in accordance with the consensus standards for acquisition, measurement, and reporting of OCT studies [19].

### Comparison of differences in coronary diameter Z-score and cross-sectional intimal area

We compared differences in UCG-determined coronary artery diameter Z-scores between acute (i.e., time of first CAG) and convalescent (i.e., time of OCT) phases, relative to OCT-determined coronary arterial wall structure in the convalescent phase. In accordance with the AHA guideline [15], we defined the acute phase as approximately 8 weeks after the onset of KD; we defined the convalescent phase as ≥ 5 years after the onset of KD. CAA regression was defined as a normal coronary artery diameter physique (Z-score < 2.5), combined with smooth coronary arteries on UCG and CAG.

### Statistical analysis

Quantitative variables are expressed as medians with ranges and categorical variables are expressed as numbers with percentages. Statistical analyses were performed using the Mann–Whitney U test, Fisher’s exact test, and Spearman rank correlation coefficient in JMP Pro software, version 13 (SAS Institute Japan, Tokyo, Japan). P < 0.05 was considered statistically significant.

## Results

### Patient characteristics

Twenty-three patients were included in the analysis. Patient characteristics are shown in Table 1. The median age at KD onset was 1 year and 2 months. The male:female ratio was 18:5. Twenty-one patients (91%) were treated with IVIG, and 13 patients (62%) did not respond. As second-line treatment, four patients (17%) were treated with steroids and two patients (9%) were treated with cyclosporine. The median interval between KD onset and initial CAG was 2 months. Two patients had an interval longer than 4 months (5 and 13 months, respectively) because of CAA-related myocardial infarction in the early acute phase; the other 21 patients underwent initial CAG within 3 months. The median age at OCT was 18 years and 2 months. The median interval between KD onset and OCT was 16 years and 9 months. No OCT-related complications occurred.

**Table 1:** Patients characteristics

		(Minimum–maximum)
Number of patients	23	
Male:female	18:5	
Age at the onset of KD	1 y 2 m*	(1 m–10 y 11 m)
Interval between the onset of KD and the first CAG	2 m*	(1 m–1 y 1 m)
Age at the OCT examination	18 y 2 m*	(11 y 1 m–29 y 3 m)
Interval between the onset of KD and OCT	16 y 9 m*	(5 y 1 m–24 y 4 m)

*Median

1 y 2 m: 1 year and 2 months

### Arterial branch characteristics

**Fig. 2:**
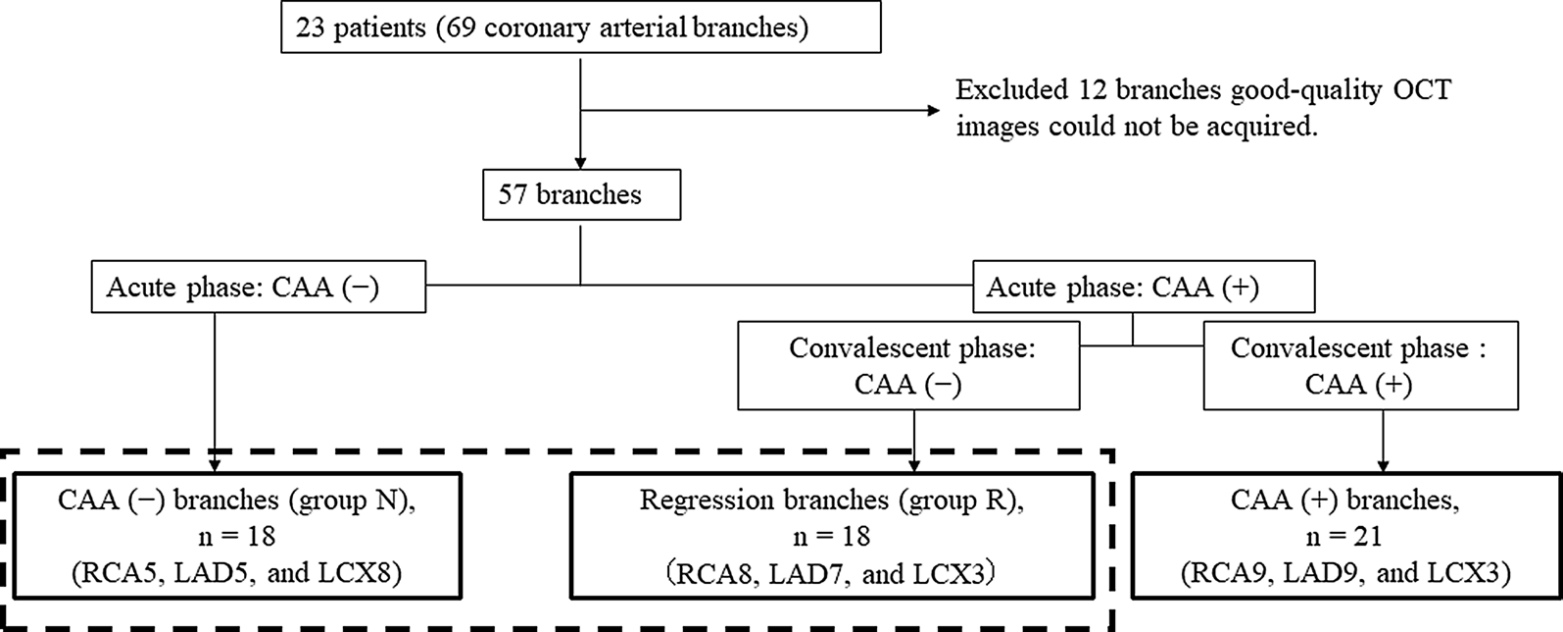
Study flowchart. KD, Kawasaki disease; CAG, coronary angiography; OCT, optical coherence tomography; CAA, coronary artery aneurysm; RCA, right coronary artery; LAD, left anterior descending coronary artery; LCX, left circumflex coronary artery

Among the 23 patients in this study, 69 coronary arterial branches were examined. However, good-quality OCT images could not be acquired in 12 branches; therefore, 57 branches were analyzed. CAAs were not found on the most recent CAG in 36 branches (63%). Eighteen of these branches were classified as group R (right coronary artery [RCA], eight; left anterior descending coronary artery [LAD], seven; and left circumflex coronary artery [LCX], three) and 18 branches were classified as group N (RCA, five; LAD, five; and LCX, eight; Fig. 2).

### Arterial wall structure changes

**Fig. 3:**
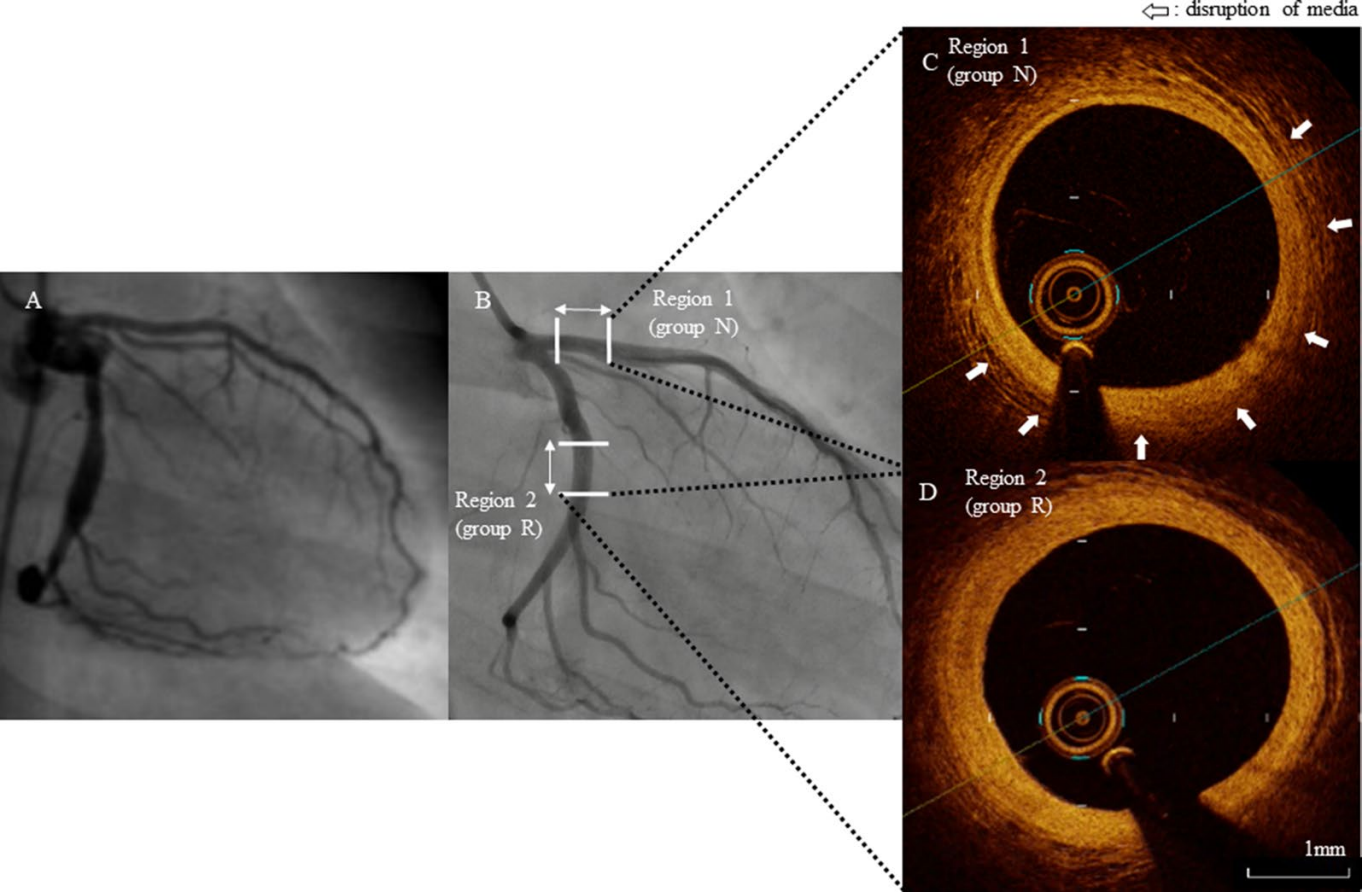
**A**, **B** Left coronary angiography in the same patient at two time points (**A** age 9 months, acute phase 53 days after onset; **B** age 12 years and 10 months, convalescent phase 12 years and 2 months after onset). **C**, **D** Optical coherence tomography (OCT) images of a coronary artery in the convalescent phase (**C**, region 1; **D**, region 2). **A** The left anterior descending coronary artery (LAD) appears normal (proximal inner diameter, 2.1 mm) and the left circumflex artery (LCX) has a small coronary artery aneurysm (CAA) (maximum inner diameter, 4.0 mm). **B** The LAD remains normal (proximal inner diameter, 3.1 mm; region 1; classified in group N) and the LCX CAA has regressed (inner diameter, 2.7 mm; region 2; classified in group R). **C** OCT of region 1 shows the three arterial lamina, partial disruption of the media (white arrows), and mild intimal thickening. **D** OCT of region 2 shows widespread abnormal intimal thickening and disruption of the media

In group R, OCT showed widespread and considerable intimal thickening with disruption of the media in the region of observation. Unexpectedly, regions of observation in group N showed mild intimal thickening and partial disruption of the media (Fig. 3).

A summary of UCG and OCT data from both groups is shown in Table 2. The median total doses of IVIG were 2600 mg/kg and 2500 mg/kg, respectively; there were no differences between groups R and N. The median maximum intimal thickness and intimal cross-sectional area were significantly greater in group R than in group N. The incidences of abnormal intimal thickening (> 400 µm) and disruption of the media were significantly higher in group R than in group N (p < 0.001 and p = 0.02, respectively). Calcification, macrophage colonization, and thrombus were not found in either group.

**Table 2:** Summary of IVIG dose, ultrasound cardiography and optical coherence tomography data in both Group R and Group N

	Group R (n = 18)	Group N (n = 18)	p value
Total dose of IVIG (mg/kg)	2600* (0–4200)	2500* (0–4000)	p = 0.820
UCG-determined coronary artery diameter Z-score in the acute phase (i.e., time of first CAG)	7.13* (3.65–13.88)	1.81* (-0.18–2.23)	p < 0.001
UCG-determined coronary artery diameter Z-score in the convalescent phase (i.e., time of OCT)	0.64* (-0.02–2.11)	0.48* (-0.67–1.60)	p = 0.311
Difference in UCG-determined coronary artery diameter Z-scores between acute and convalescent phases	6.26* (2.59–13.9)	1.14* (0.22–2.14)	p < 0.001
Maximum intimal thickness (µm)	475* (386–646)	355* (118–552)	p = 0.007
Number of abnormal intimal thickening (> 400 µm), n (%)	17 (94)	7 (39)	p < 0.001
Intimal cross-sectional area (mm^2^)	4.12* (2.04–7.86)	2.88* (1.45–5.70)	p = 0.029
Disruption of the media, n (%)	18 (100)	12 (67)	p = 0.020

*IVIG* intravenous immunoglobulin, *CA* coronary artery, *CAG* coronary angiography, *OCT* optical coherence tomography, *UCG* ultrasound cardiography, *KD* Kawasaki disease

*Median (minimum–maximum)

### The correlation between the difference in Z-score of the coronary artery diameter according to UCG and wall structure according to OCT

**Fig. 4:**
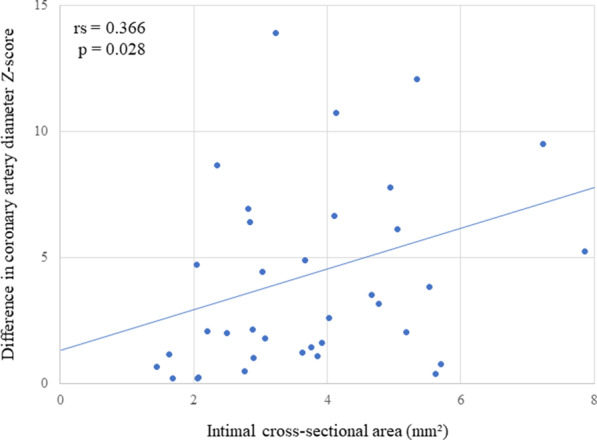
Correlation between differences in coronary artery diameter Z-score and intimal cross-sectional area. The median difference in UCG-determined coronary artery diameter Z-score between acute and convalescent phase was 2.37 (range 0.22–13.90). OCT examination revealed intimal thickening in many patients, and there was a correlation between the difference in UCG-determined coronary artery diameter Z-score between acute and convalescent phases and OCT-determined intimal cross-sectional area (rs = 0.366, 95% confidence interval: − 0.219 to 0.579; p = 0.028)

The median difference in UCG-determined coronary artery diameter Z-score between acute and convalescent phases was 2.37 (range, 0.22–13.90); the Z-scores of all coronary arteries were lower in the convalescent phase than in the acute phase, regardless of subsequent CAA development and otherwise normal appearance. The difference in coronary artery diameter Z-score was correlated with the intimal cross-sectional area (rs = 0.366, 95% confidence interval − 0.219 to 0.579; p = 0.028) (Fig. 4).

## Discussion

This study used OCT to examine coronary arterial wall changes in 23 patients who developed CAA in the acute phase of KD. We compared structural changes in the three layers of the arterial wall between arterial branches without CAA in either the acute or convalescent phases (group N) and arterial branches with CAA in the acute phase that regressed in the convalescent phase (group R). Intimal thickening and (partial) disruption of the media were found in the coronary arterial walls in both groups R and N. To the best of our knowledge, this is the first study to use OCT to identify intimal thickening and disruption of the media in coronary arterial walls damaged by inflammation caused by KD, including in arteries not affected by CAA development.

Many autopsy studies have reported various arterial wall changes that occur in arterial segments associated with CAA, such as intimal thickening, disruption of the media, and calcification [20]. In addition, these changes can be found in arteries where the CAA regressed in the convalescent phase, which resulted in normal arterial appearance on UCG and selective CAG [5].

The three layers of the coronary arterial wall (intima, media, and adventitia) can be clearly distinguished using OCT. We found that all group R arterial branches exhibited abnormal intimal thickening (> 400 µm). In a study using IVUS, Tsuda et al. [13] also found that intimal–medial thickening > 400 µm frequently developed in coronary branches that dilated to > 4.0 mm diameter within 100 days of KD onset. In addition, Dionne et al. [9] reported that the mean intimal thicknesses in segments with a CAA were 315 µm in the RCA, 455 µm in the LAD, and 360 µm in the LCX.

Abnormal intimal thickening develops in the involved arterial area after CAA regression, although the inner diameter appears normal on echocardiography and CAG. However, endothelial cell function is not normal in these remodeling vessels, as shown by a previous study [21]. Intimal dysfunction in the area of CAA regression may induce local stenosis or arteriosclerosis in the future.

The incidence of abnormal intimal thickening (≥ 400 µm) in coronary arterial branches was lower in group N (39%) than in group R (100%); however, the median maximum intimal thickness in group N was 335 µm (range, 118–552), which is significantly greater than normal. These data suggest that KD-related inflammation may affect both CAA-associated branches and branches without CAA. Therefore, coronary arterial branches with normal characteristics in the acute phase may later develop intimal thickening and disruption of the media. Several autopsy studies have reported KD-related inflammatory damage in the walls of coronary arteries not affected by coronary artery lesions [22, 23], which supports our findings.

Intimal thickening in coronary arterial branches unaffected by CAA is an important consideration. The median maximum intimal thickness in group N in our study was 355 µm; however, Dionne et al. reported a median maximum intimal thickness of 61.7 ± 17 µm [9]. Differences between the two studies in the evaluated segments of the coronary arteries may explain this discrepancy. Our study evaluated only a 10-mm segment of the proximal coronary arterial branches in group N, while Dionne et al. did not specify the region of evaluation. We selected a proximal segment for two reasons. First, this area is easily evaluated by OCT; second, the evaluated segments needed to be equivalent between groups.

In several reports, KD patients with coronary artery lesions that regressed and became angiographically normal later developed cardiovascular disorders at a young age [24, 25]. Other studies have reported that the coronary arterial wall in segments unaffected by aneurysms has abnormal vascular function similar to the function observed in segments associated with regressed lesions [26–28]. These studies support our findings. Notably, KD patients with normal appearing coronary arteries in the acute phase may later develop various cardiovascular disorders, similar to patients who develop CAAs that later regress.

This study had several limitations. First, several biases were present, such as the age at KD onset and interval between KD onset and OCT. Adjustments regarding these factors were not performed because of the limited number of patients. Second, we could not perform OCT in KD patients who did not develop CAA in all three branches because of ethical considerations; therefore, data regarding intimal thickness and disruption of the media in these patients was not collected. Third, although differences regarding additional treatment may affect intimal thickening and media disruption, the number of patients with additional treatment was insufficient for subgroup comparisons in this study. Last, selection bias involving individual differences in coronary artery damage among KD patients could not be completely eliminated.

## Conclusions

In KD patients, intimal thickening and disruption of the media occur in coronary arteries with acute phase CAAs that later regress in the convalescent phase, as well as in arteries with normal CAG findings in the acute and convalescent phases. If these changes are associated with healing from KD vasculitis, long-term follow-up of KD patients may be required, regardless of CAA status.

## Data Availability

The datasets used and analyzed during the current study are available from the corresponding author on reasonable request.
